# Evaluation of HSV-2 gE Binding to IgG-Fc and Application for Vaccine Development

**DOI:** 10.3390/vaccines10020184

**Published:** 2022-01-25

**Authors:** Jennifer D. Galli, Melanie Horton, Eberhard Durr, Gwendolyn J. Heidecker, Daniel Freed, Arthur Fridman, Dai Wang, Lan Zhang

**Affiliations:** 1 Infectious Diseases and Vaccines Discovery, Merck & Co., Inc., West Point, PA 19486, USA; melanie_horton@merck.com (M.H.); eberhard.durr@merck.com (E.D.); gwendolyn_heidecker@merck.com (G.J.H.); dan_freed@merck.com (D.F.); dai_wang@merck.com (D.W.); lan_zhang2@merck.com (L.Z.); 2 Data Science and Scientific Informatics, Merck & Co., Inc., Rahway, NJ 07065, USA; arthur_fridman@merck.com

**Keywords:** herpes simplex virus, glycoprotein, vaccine, HSV, gE, gI, heterodimer, antibody

## Abstract

Glycoprotein E (gE) and glycoprotein I (gI) are expressed as a heterodimer on the surface of Herpes simplex virus (HSV). Glycoprotein E binds Fc domain of immunoglobulin G (IgG) and inhibits activities mediated by the IgG Fc domain, contributing to immune evasion by HSV. It has been reported that HSV type 1 gE (gE-1) is capable of binding IgG Fc as a monomer and in a heterodimeric complex with gI, with the heterodimer having 50- to100-fold greater affinity for Fc than gE alone. We report the production of both a soluble form of HSV type 2 gE (gE-2) and a soluble HSV-2 gE/gI heterodimer (gE-2/gI-2). Characterization of soluble gE-2 by surface plasmon resonance (SPR) demonstrates that it is incapable of binding human IgG or the IgG Fc domain. Co-expression with HSV-2 gI (gI-2) and purification of the gE-2/gI-2 heterodimer enable gE-2 to bind human IgG through its Fc domain. We hypothesize that functional epitopes of wildtype gE-2 may be masked by plasma IgG Fc and affect the immunogenicity of the gE-2/gI-2 heterodimer as a vaccine antigen. A series of gE-2 mutations within the surface-exposed Fc:gE-2 interface was designed, and gE-2 mutants were co-expressed with gI-2. Evaluation of twelve gE-2 mutant heterodimers by SPR assay identified nine gE-2 mutations which abrogated or reduced Fc binding while maintaining heterodimer formation with gI. Vaccinating rabbits with the four most Fc-binding deficient gE-2/gI-2 heterodimers elicited comparable anti-heterodimer binding antibody titers and statistically significantly higher serum neutralization antibody levels than wildtype heterodimers. Taken together, these data support the concept of rational antigen design for improved vaccine candidates.

## 1. Introduction

Herpes simplex virus type 2 (HSV-2) is an enveloped, double-stranded DNA virus and is the primary cause of genital ulcers in the United States [1]. A distinguishing feature of herpesviruses, HSV-2 included, is the establishment of a latent infection by the virus in peripheral nervous system neurons. Despite a primed immune system, reactivation of the virus is frequent, often leading to lesions at the original site of infection.

The HSV-2 genome encodes multiple glycoproteins that are expressed on the virus surface. Glycoprotein E (gE) and glycoprotein I (gI) are expressed as a heterodimer on the surface of virions and infected cells [2]. The gE/gI heterodimer is responsible for cell-to-cell spread of the virus [3,4]. Importantly, glycoprotein E also plays a role in immune evasion of the virus. It has also been shown that gE binds to the Fc domain of immunoglobulin G (IgG) and inhibits immunologic activities facilitated by the IgG Fc domain through a process described as antibody bipolar bridging [2,5,6,7]. This binding activity has been shown to shield and protect the virus from Fc-mediated immune responses, including viral neutralization and antibody-dependent cellular cytotoxicity [5,8].

**Figure 1:**
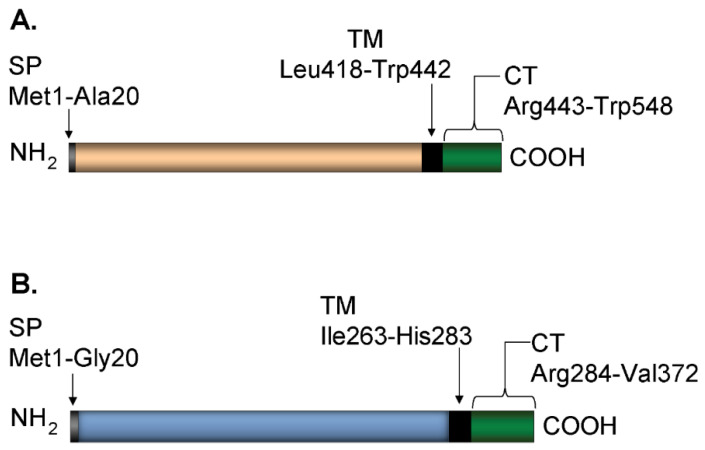
Schematic diagram of gE-2 and gI-2. Schematic diagram of the domain structure of wildtype gE-2 (**A**) and wildtype gI-2 (**B**) from strain 333. SP: signal peptide, TM: transmembrane domain, CT: cytoplasmic tail. Domain boundaries denoted with amino acid numbers. Domain architecture identical for all strains of HSV-1 and HSV-2 gE and gI. Total amino acid length varies among HSV type and strain.

The gE-2/gI-2 heterodimer comprises two viral glycoproteins. HSV-2 gE is an approximately 550 amino acid type I membrane glycoprotein. Its domain architecture includes a ~440 amino acid extracellular domain, followed by a transmembrane segment and a ~100 residue cytoplasmic tail (Figure 1A). HSV-2 gI is the smaller protein in the heterodimer having ~370 amino acids. HSV-2 gI is also a type I transmembrane protein with a ~280 amino acid extracellular domain and a ~90-residue cytoplasmic tail (Figure 1B). The cytoplasmic tails of HSV gE-1/gI-1 and related proteins have been shown to contain motifs that target transportation of membrane proteins to the trans-Golgi network and lead to endocytosis of membrane proteins from the plasma membrane [9,10,11,12]. Literature precedents have shown that the soluble extracellular domain of gE-1 is capable of binding to IgG Fc both alone and when complexed with soluble gI-1, and the cytoplasmic tails of gE-1 and gI-1 are not required for binding to IgG Fc [7,13,14,15,16].

Several studies have been published that elucidate details of the interaction between HSV-1 gE/gI heterodimer (gE-1/gI-1) and IgG Fc. While gE-1 is capable of associating with Fc alone, the gE-1/gI-1 heterodimer has been shown to have 50- to 100-fold greater affinity for IgG Fc than gE-1 alone [13]. HSV-1 gI itself does not bind IgG, indicating that the heterodimer:Fc direct interacting residues are likely within gE [16]. Indeed, it has been shown that the residues responsible for Fc binding lie within the C-terminal portion of the extracellular domain of gE-1, while those that associate with gI-1, forming the heterodimer interface, are within the N-terminal portion of the gE-1 [14,15,17,18]. Johansson and colleagues demonstrated that HSV-1 infected cells interact with rabbit and human IgG but not with rodent IgG, showing that the interaction is species dependent [19]. The binding affinity between HSV-1 infected cells and IgG also depends on the IgG isotype, with Kd values for IgG4 being the highest and IgG3 being the least among the human isotypes [20,21,22].

While basic research on HSV biology has centered around the glycoproteins of HSV-1, vaccine efforts have focused on HSV-2 owing to its association with recurrent genital lesions. Two previous vaccine trials were conducted in which participants were vaccinated with viral glycoprotein gD-2 alone [23,24], or in combination with gB-2 [25]. Improvements upon these vaccines have been attempted with the addition of glycoproteins gC-2 and gE-2, which are involved in HSV immune evasion, using both protein subunit and mRNA vaccine modalities [26,27,28,29,30]. These combination vaccines have shown promise in mouse and guinea pig HSV viral challenge models but have not yet been introduced in human clinical trials. A commonality among these vaccine candidates is the use of wildtype protein sequence for vaccine antigens and lack of structural or functional engineering of the antigens. As HSV gE mediates immune evasion through its interaction with IgG Fc, we postulate that a gE vaccine antigen that induces anti-gE antibodies and effectively blocks the interaction between HSV gE and IgG Fc would abrogate this immune evasion. A potential concern of using wildtype gE protein as the vaccine antigen is that antigenic epitopes of wildtype gE could be masked by a non-specific host IgG shield decorating gE through binding of the Fc domains. This “shield” would render gE epitopes inaccessible to the immune system, decreasing the specific gE antibody response. This is especially concerning as the IgG concentration in human serum is very high, about 8–16 mg/mL (~50–100 µM) [31,32]. Previous studies on mutant fHbp (human factor H binding protein) vaccines against *Neisseria meningitidis* have established a proof of concept that antigens with artificially lowered host protein binding could elicit higher and broader functional antibody responses than wildtype antigens [33]. We hypothesize that an engineered gE antigen that does not bind IgG Fc would eliminate the IgG shielding of gE and leave surface residues exposed, thereby allowing access for the immune system to the antigenic sites of gE, resulting in an improved antibody response.

The purpose of this study is to evaluate binding of host IgG to gE-2 and gE-2/gI-2 heterodimers, including potential species specificity, and to use these data in combination with homology modeling to design improved antigens for vaccine development. To gain insight into the properties and function of HSV-2 glycoproteins gE-2 and gI-2, we explored the ability of gE-2 to associate with IgG Fc either alone or as part of the heterodimer with gI-2. Here, we report the production of both a soluble form of gE-2 and a soluble gE-2/gI-2 heterodimer in mammalian cells. We then characterized the association of IgG Fc from different species with gE-2 monomer and gE-2/gI-2 heterodimer using surface plasmon resonance (SPR). We designed a series of mutations on gE-2 within the hypothesized Fc:gE-2 interface based on the published gE-1:Fc crystal structure [14] and characterized these gE-2 mutant heterodimers for IgG Fc binding by SPR. Finally, we evaluated four Fc-binding deficient gE-2/gI-2 mutant heterodimers in an in vivo immunogenicity study to determine their ability to elicit an improved immune response.

## 2. Materials and Methods

### 2.1. Plasmid Construction

Protein sequences for mammalian expression of gE-2 t417, gE-2 t405, gE-2 t24-405, and gI-2 t262 were derived from HSV-2 strain 333 and truncated prior to the transmembrane domain at the indicated amino acid numbers, where amino acid 1 denotes the starting methionine in the signal peptide (Figure 1). Additionally, the native signal peptide (amino acids 1–23) of gE-2 t24-405 was replaced by the mouse Igκ signal peptide. Protein sequences of gE-2 t414 and gI-2 t262 were derived from HSV-2 strain HG52 and truncated prior to the transmembrane domain at the indicated amino acid. HSV-1 protein sequences of gE-1 t419 and gI-1 t269 were derived from strain KOS and truncated prior to the transmembrane domain at the indicated amino acid. The gE-2 mutant sequences #1–#15 were made in the gE-2 t417 parental plasmid background with point mutations for amino acids indicated in Table 1. All protein sequences for mammalian expression were cloned into the pV1JNS vector, codon-optimized for human cell-line expression by Genewiz (South Plainfield, NJ, USA), and included a double glycine linker, thrombin cleavage site, and either a 6-histine tag (gE constructs) or FLAG tag (gI constructs). For insect cell expression, Bac gE-1 t411 was inserted into the multiple cloning site (MCS) of pFastBac1 by cloning HSV-1 strain 17 derived gE-1 amino acids 26 to 411 with a triple glycine linker and 8-histidine tag at the 3′ end prior to the stop codon. This cloning strategy also includes replacement of the native signal peptide (amino acids 1–25) with the honeybee melittin signal peptide and inclusion of an extra alanine following the new signal peptide.

### 2.2. Protein Expression

For mammalian cell-expressed proteins, expression plasmids were transfected either singly (gE) or co-transfected (gE-gI) into Expi293 cells (ThermoFisher, Waltham, MA, USA) using Expifectamine (ThermoFisher) following the manufacturer’s recommended protocol. Cell supernatants were harvested 72 h post-transfection and clarified by centrifugation at 10,700× *g* at 20 °C for 30 min. Clarified supernatants were aliquoted into 250 mL Corning bottles and transferred to −70 °C for storage until purification. For Bac gE-1 t411 expressed in insect cells, Sf9 insect cells were infected with P1 baculovirus stock at a 1:10,000 dilution at LakePharma (formerly Blue Sky Biotech, Inc., Worcester, MA, USA), Worcester, MA. Insect cell supernatants were clarified by centrifugation prior to protein purification.

### 2.3. Purification of Soluble gE and gE/gI Heterodimers

For purification of mammalian cell-expressed protein, the gE and gE/gI heterodimers were purified from clarified Expi-293 cell supernatants. The supernatants were adjusted by addition of 50 mM HEPES, pH 7.5, and 150 mM NaCl. Adjusted supernatants were purified using a HisTrap Ni Sepharose column (Cytiva, Marlborough, MA, USA) and eluted with 300 mM imidazole. Protein-containing fractions were pooled, further purified, and buffer exchanged (10 mM HEPES, pH 7.5, 150 mM NaCl) by size-exclusion chromatography (SEC) on a Superdex 200 column (Cytiva). Protein-containing fractions were pooled and concentrated using a 10 kDa molecular weight cutoff Ultrafree-15 centrifugal filter before addition of 0.005% PS-20. For insect cell-expressed gE1-t411, Bac gE-1 411 harvested conditioned medium was subjected to tangential flow filtration (MWCO 10 kDa) for concentration and simultaneous buffer exchange into 1X phosphate-buffered saline (PBS) at pH 7.4. The material was applied to Ni-Sepharose resin column (Cytiva) at 4 °C and washed three times, with washes 2 and 3 containing 20 mM imidazole and 50 mM imidazole, respectively. Bound protein was eluted using a linear imidazole gradient. Prominent Bac gE-1 t411 containing fractions were pooled and dialyzed into 25 mM HEPES, pH 7.5 and 9% sucrose. Final protein concentration was determined by A280 analysis using protein-specific reduced extinction coefficients calculated from amino acid sequence. Protein purity was estimated by Coomassie-stained gel containing a 3-fold dilution series across 6 lanes with a starting concentration of 1 μg/μL in a 5 μL load volume.

### 2.4. SDS-PAGE and Western Blot

A total of 400 µg of each protein was resolved on a 4–12% gradient, 1.5 mm, 15-well Bis-Tris NuPAGE gel in 1X MES buffer (Invitrogen, ThermoFisher). SeeBlue Plus2 pre-stained protein standard (ThermoFisher) was included on the SDS-PAGE for molecular weight reference. Proteins were transferred onto nitrocellulose membranes using the iBlot Western blotting system (Invitrogen, ThermoFisher). Membranes were blocked overnight at 4 °C with 50 mL of 5% non-fat dry milk (Blotting-grade blocker, Bio-Rad Laboratories, Hercules, CA, USA) in 1X tris-buffered saline containing 0.1% Tween-20 (1X TBST). Membranes were probed with either anti-FLAG-alkaline phosphatase antibody (Sigma, A9469, St. Louis, MO, USA) or anti-polyhistidine-alkaline phosphatase antibody (Sigma-Aldrich, catalog #A5588) diluted 1:1000 in 5% non-fat dry milk in 1X TBST for 1 h at 4 °C. Membranes were washed 3 times for 5 min each at room temperature with 1X TBST. Membranes were developed using 1-step NBT/BCIP (ThermoFisher) according to the manufacturer’s recommendations.

### 2.5. Surface Plasmon Resonance

All experiments were performed on a Biacore 2000 (Cytiva). ChromePure human IgG Fc and human, rabbit, mouse, and guinea pig whole molecule IgG (Jackson ImmunoResearch Inc., West Grove, PA, USA) were amine coupled onto CM5 sensor chips (Cytive) at 854, 1348, 1094, 857, and 1143 RU, respectively, using manufacturer recommended conditions. HSV gE monomers and gE/gI heterodimers were diluted to 750 μg/mL (except Bac gE-1 t411 at 490 μg/mL) in HEPES-buffered saline with surfactant (0.01 M HEPES pH 7.4, 0.15 M NaCl, 0.005% *v*/*v* Surfactant P20, Cytiva) and flowed at 30 μL/min over the IgG sensor surface. The surface was regenerated after each cycle with 0.25 M di-ammonium citrate, pH 5.0. Data were analyzed using BIAevaluation version 4.1.1 software. All data were background subtracted with a flow cell having no IgG surface-coupled and flowed over with matching protein.

### 2.6. Size Exclusion Chromatography/Multi-Angle Laser Light Scattering

Separation of HSV gE monomer or gE/gI heterodimer protein complex was performed on an Agilent 1100 HPLC system consisting of a quaternary pump, a vacuum degasser, a thermostated autosampler, and column compartment with a Superdex 200 30/100 GL column (Cytiva). The column was kept at room temperature (20 °C). The mobile phase (10 mM HEPES, 150 mM NaCl, pH 7.5) was pumped at 0.5 mL/min. To reduce baseline noise in the light scattering detector, a 25 mm high-pressure filter with 0.1 µm pores (MilliporeSigma, Burlington, MA, USA) was used for in-line filtration of the mobile phase. Detection was carried out using the Agilent HPLC DAD UV-detector G1315B (280 nm) and Wyatt Technology’s (Santa Barbara, CA, USA) DAWN Heleos II light-scattering and T-Rex refractive index detectors. The refractive index increment values (dn/dc) of 0.185 mL/g for protein and 0.134 mL/g for carbohydrate were used for molecular weight calculations. Protein UV extinction coefficients were calculated based on the primary sequence using Vector NTI Advance 11 (Invitrogen, Waltham, MA, USA). The Dawn Heleos II detector was calibrated with toluene according to the manufacturer’s instructions. A total of 200 µg of protein complex sample was injected for each analysis. Determination of protein mass content and molecular weight was performed by the ASTRA 6.1 software (Wyatt Technology, Santa Barbara, CA, USA) using the conjugate analysis algorithm. More details on the use of three detector systems for the analysis of protein complexes were reported elsewhere [34,35].

### 2.7. Rabbit Immunization Study

New Zealand White female rabbits of 3 to 4 months of age were obtained from a specific pathogen-free colony (Labcorp Drug Development, Princeton, NJ, USA). Animals were housed individually in a Merck animal facility, in accordance with the Guide for the Care and Use of Laboratory Animals, and the facility is credentialed by the Association for Assessment and Accreditation of Laboratory Animal Care [36]. Groups of rabbits (N = 4) were immunized at weeks 0, 3, and 8 with intramuscular injections of 20 μg of various proteins in the presence of 90 μg Adju-Phos (Brenntag, Ballerup, Denmark), as well as a control group of Adju-Phos alone in 0.5 mL saline, and the immune sera were collected at weeks 0, 5, and 10.

### 2.8. Antibody ELISA Assay

NUNC Maxisorb plates were coated with gE-2/gI-2 heterodimer antigen at 2.0 μg/mL in PBS with 50 μL per well and incubated at 4 °C overnight. Plates were washed with PBS with 0.05%Tween-20 (PBST) and blocked with PBST containing 3% nonfat dry milk for 1 h at room temperature with gentle rocking. Serial diluted samples were added to each well in duplicates, and the plates were incubated at room temperature for 1 h with rocking. Following incubation, the plates were washed 6 times and incubated for 1 h with HRP-conjugated secondary Ab (Goat anti-Rabbit IgG (H+L)-HRP Southern Biotech, Birmingham, AL, USA) at room temperature. Plates were developed colorimetrically by 5–10 min incubation with TMB substrate (Virolabs, Chantilly, VA, USA) at room temperature in the dark. The reaction was stopped with addition of 1N H_2_SO_4_. Plates were read for absorbance at 450 nm on a VersaMax microplate reader. The interpolated endpoint titers were calculated by linear interpolation of data between the dilutions whose responses bracket the designated cut-off of 2.5x the median of the blank wells [37,38].

### 2.9. Neutralization Assay

U-2 OS cells were seeded in a 96-well culture plate at 5 × 10^4^ cells per well and grown at 37 °C with 5% CO_2_ for 18–24 h. Serum samples were diluted initially at 1:10 in sample diluent (U-2 OS growth media containing 2% FBS) containing 5% baby rabbit complement followed by seven serial 4-fold dilutions. HSV-2 N2 lacZ reporter virus [39] was diluted in sample diluent containing 5% baby rabbit complement to 2.5 × 10^4^ pfu/mL, added to each well, and incubated for 1 h on a rotary shaker at 37 °C with 5% CO_2_. For neutralization assays completed in the presence of non-specific IgG, HSV-2 N2 lacZ virus stock was pre-incubated with 10 mg/mL of rabbit IgG (MilliporeSigma, catalog # I5006) for 1 h at 37 °C, then diluted as described above. Following incubation, supernatant was aspirated from cell-containing wells. A total of 200 µL of virus/sample mixture was overlaid and incubated 18–24 h at 37 °C with 5% CO_2_. Subsequently, supernatant was aspirated from all wells, 50 µL/well of lysis buffer (PBS with 0.2% Triton X-100) was added, and plates were incubated at room temperature for 10 min with rocking. Following lysis, 200 µL of development substrate (75 mM Na_2_PO_4_, 32 mM NaHPO_4_, 5 mM KCl, 2 mM MgSO_4_, 50 µM β-mercaptoethanol, and 0.5 mM chlorophenol red-β-D-galactosidase) was added to each well, incubated at room temperature for 15–30 min, and OD_562_ was read on a VERSAmax microplate reader (Molecular Devices, San Jose, CA, USA). Included on each plate were cell control wells containing no virus, representing 100% neutralization, and virus control wells containing no sample, representing 0% neutralization. ODs were converted to % neutralization, and NT50s were determined using GraphPad Prism via non-linear curve fit log (inhibitor) vs. response, variable slope (four parameters), with bottom constrained at 0 and top at 100. Statistical significance was determined by two-way ANOVA and Tukey’s multiple comparisons test where P < 0.05.

## 3. Results

### 3.1. Soluble gE-2 Forms a Heterodimer with Soluble gI-2 When Co-Transfected into Mammalian Cells

**Figure 2:**
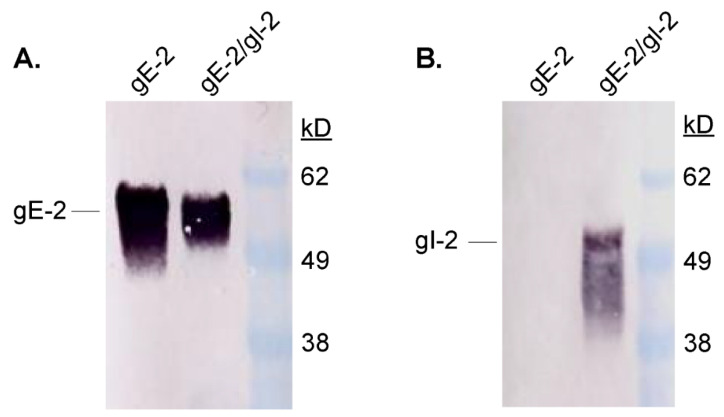
Purified gE-2 monomer and gE-2/gI-2 heterodimer protein from transiently transfected Expi293 cells. Cells were transfected with either gE-2 His-tagged expression plasmid alone or co-transfected with gE-2 His-tagged and gI-2 FLAG-tagged expression plasmids. Clarified supernatant was purified by anti-His affinity chromatography and SEC. Purified protein was separated via SDS-PAGE, blotted onto membranes, and probed with either anti-His (**A**) or anti-FLAG (**B**) antibodies. The positions of gE-2 and gI-2 proteins and marker proteins in kilodalton (kDa) are indicated.

We produced a soluble form of gE-2 (Figure 1A) and a soluble gE-2/gI-2 heterodimer (Figure 1A,B) in mammalian cells. Both the monomer gE-2 and heterodimer gE-2/gI-2 were purified by affinity and size exclusion chromatography. In order to easily distinguish the monomer gE-2 and gI-2 proteins, we tagged the gE-2 with 6x histidine and gI-2 with FLAG. Western blot analysis indicates a single his-tag reactive band for the purified gE-2 alone (Figure 2A). In contrast, reactive bands are present to both the gE-2 (anti-his tag) and gI-2 (anti-FLAG) proteins from the co-transfected samples (Figure 2A,B), suggesting the presence of a heterodimer as the purification was based on affinity purification of the histidine-tagged gE-2. The average purified protein yield of the gE-2/gI-2 heterodimer was 103 mg/L with an estimated purity range of 87–90% by Coomassie-stained gel. To further characterize the gE-2 monomer and gE-2/gI-2 heterodimer complex, we performed a molecular weight analysis by SEC-MALS (Table 2, lines 1–9). The observed molecular weight of soluble gE-2 was 49.9 kDa, which corresponds to the expected molecular weight of a monomer (Table 2, line 3). The observed molecular weight of the heterodimer, 69.9 kDa, confirms a 1:1 ratio of soluble gE-2 to gI-2 in the complex (Table 2, line 6).

### 3.2. gE-2 Requires gI-2 to Bind IgG Fc

**Figure 3:**
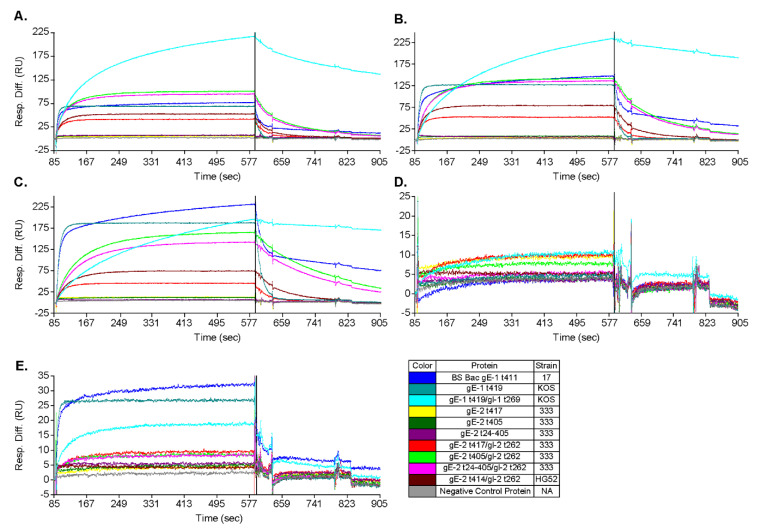
Binding of gE-2 monomer and gE-2/gI-2 heterodimer to IgG Fc: SPR analysis. gE monomers and gE/gI heterodimers were flowed over (**A**) human IgG Fc and (**B**) human, (**C**) rabbit, (**D**) mouse, and (**E**) guinea pig whole-molecule IgG coupled to CM5 chips. gE-1 monomers and gE-1/gI-1 heterodimer are included as positive controls. A protein that does not associate with IgG Fc is included as a negative control. Black line denotes transition from association phase to dissociation phase.

Works by Sprague, Johnson, and others have shown that gE-1 is capable of binding IgG Fc both alone and when complexed with gI-1, with the heterodimer having a ~100-fold greater affinity for IgG Fc [7,13,17]. We confirmed by SPR that soluble gE-1 from two different strains, KOS and 17, and two different truncation points all associated with human IgG Fc and whole IgG, regardless of whether in the monomer or gE/gI heterodimer form (Figure 3A,B and Table 3 lines 1–3). Similarly, we characterized IgG binding of the gE-2 monomer and gE-2/gI-2 heterodimers from two strains and two truncation points by SPR. Interestingly and unlike gE-1, soluble gE-2 alone is incapable of binding to human IgG or the Fc domain of human IgG, regardless of their strain backgrounds and truncation positions (Figure 3A,B, Table 3 lines 4–6). The gE-2/gI-2 heterodimer complex on the other hand is able to bind human IgG and its Fc domain (Figure 3A,B and Table 3 lines 7–10).

### 3.3. gE-2/gI-2 Heterodimers Appear to Have Different Association/Dissociation Profiles than gE-1/gI-1 Heterodimers

The curvature profile of our SPR data suggests a potential rate difference between gE-1/gI-1 and gE-2/gI-2 heterodimers when binding human whole-molecule IgG. Having set the analyte concentration equal among heterodimers and assuming an equivalent number of free ligand sites on each channel surface, the initial incline portion of the association curve depends on the association rate constant. For human whole-molecule IgG, gE-1/gI-1 heterodimers trend toward a comparatively slower apparent association than gE-2/gI-2 heterodimers (Figure 3B and Table 3). Even more striking are the apparent dissociation profiles. Based on the shape of the dissociation curve for human whole-molecule IgG, gE-1/gI-1 heterodimers trend toward a comparatively slower apparent dissociation than gE-2/gI-2 heterodimers (Figure 3B). At the end of the dissociation phase, the dissociation level decrease from the end of the association phase for gE-1/gI-1 heterodimers was 19%, whereas the level decrease for gE-2/gI-2 heterodimers ranged from 90 to 100% (Table 3).

### 3.4. gE-2 Association with IgG Fc Is Modulated by the Length of Truncated gE, HSV Strain, and Species of IgG

Differences of IgG Fc binding can be impacted by length of the soluble gE-2 protein within the heterodimer. Truncation of gE-2 (strain 333) at amino acid 405 increased association (higher RU amplitude) to IgG Fc when heterodimerized with gI-2 as compared to truncation at amino acid position 417, which is immediately adjacent to the transmembrane domain (Figure 3B light green vs. red trace and Table 3 line 7 vs. line 8).

Furthermore, IgG Fc binding also depends on the HSV strain and thereby on the amino acid sequence of gE-2. The strain HG52 gE-2 t414/ gI-2 heterodimer shows increased association (RU amplitude) to human and rabbit IgG Fc when compared to its strain 333 counterpart, where gE is truncated at an analogous amino acid position to 417 (Figure 3B,C red vs. dark red trace and Table 3 line 7 vs. 10). The endpoint amplitude (response unit: RU) of association of gE-2 to whole-molecule human and rabbit IgG as well as human IgG Fc are robust within the same experiment, thus allowing for distinguishing subtle differences among various gE-2 analytes (Table 3). The RU of association for gE-2/gI-2 of strain HG52 increased over gE-2/gI-2 of strain 333 by 1.5 fold in these species (Figure 3A–C red vs. dark red trace and Table 3 line 7 vs. 10). These observed differences, while significance is undetermined, are repeatable across multiple SPR experiments.

The gE-2 proteins evaluated were all from a mammalian expression system, most closely approximating a natural human infection. The control protein gE-1 was evaluated from both mammalian and insect cell expression systems. Mammalian-expressed gE-1t 419 strain KOS monomer shows similar binding (RU amplitude) to human and rabbit IgG as insect cell-expressed gE-1t 411 strain 17 monomer (Figure 3B,C blue vs. cyan trace and Table 3 line 1 vs. 2).

Binding of gE-2/gI-2 heterodimers to IgG Fc varied depending on the virus strain and IgG species evaluated. The binding affinity of IgG to gE-2/gI-2 (strain 333) was compared among human, rabbit, guinea pig, and mouse IgGs. The associations of gE-2/gI-2 to human and rabbit IgG are strong, with a well-defined association curve and high RU amplitudes post-association (Figure 3B,C red trace and Table 3 line 7). The association of gE-2/gI-2 to guinea pig is more difficult to define. While the initial association slopes of three of the gE-2/gI-2 heterodimers trend toward association, the amplitude of the RU response is too low to define positive association (Figure 3E red, pink, and light green traces). No association by any gE-1 or gE-2 form is observed with mouse IgG (Figure 3D and Table 3). These data are consistent with previously published data for the association of HSV-1 infected cells with various species of IgG [19]. As for gE-1, the gE-1/gI-1 heterodimer significantly increased the binding of gE-1 to human IgG but not rabbit IgG (Figure 3B,C cyan vs. light blue trace and Table 3 line 2 vs. 3). Instead, the gE-1/gI-1 heterodimerization changed the shape of the rabbit IgG association curve, suggesting a possible difference in rate constants or manner of interface interaction for this Fc species when gE-1 is complexed with gI-1 versus gE-1 alone. Both gE-1 and the gE-1/gI-1 heterodimer associated with guinea pig IgG, having a four- to twelve-fold lower overall RU amplitude when compared with human and rabbit IgGs (Table 3, line 3 vs. lines 1 and 2).

### 3.5. Mutations within the Putative gE-2:IgG Fc Interface Can Disrupt the Ability of gE-2 to Bind IgG Fc

**Figure 4:**
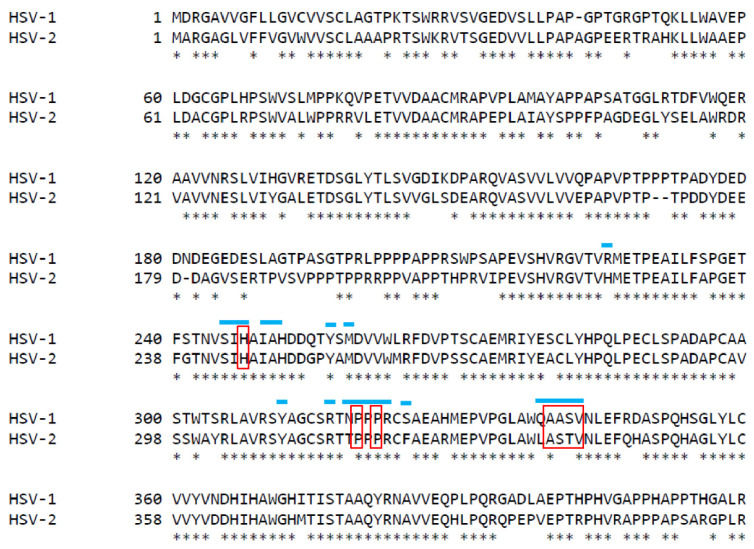
Sequence alignment of the ectodomains of gE-1 strain KOS and gE-2 strain 333. Positions targeted for mutation in gE-2 are indicated by a red box. Residues within gE-1 predicted to make direct contact with IgG Fc, based on the published co-crystal structure [14], are denoted by blue lines above the sequence.

**Figure 5:**
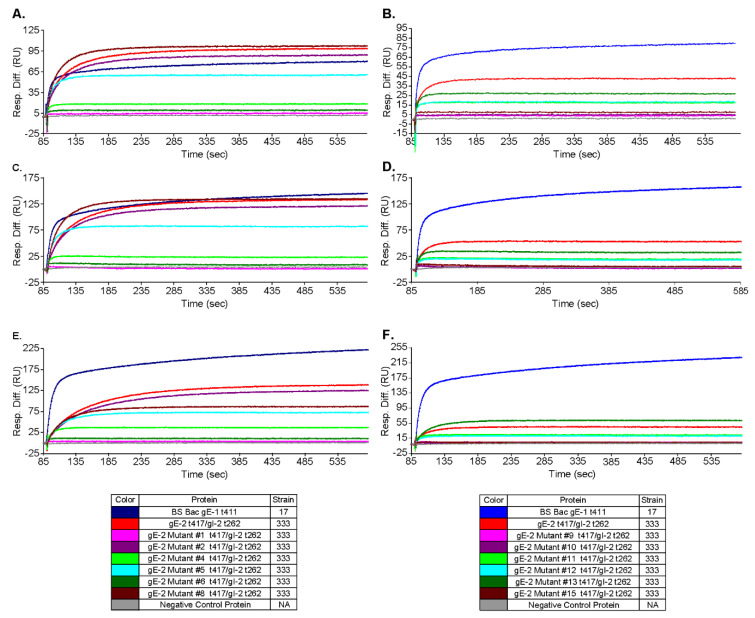
Fc binding of gE-2 mutants: SPR analysis. gE-2/gI-2 wildtype control and gE-2 mutant/gI-2 heterodimers were flowed over (**A**,**B**) human IgG Fc, (**C**,**D**) human, and (**E**,**F**) rabbit whole-molecule IgG coupled to CM5 chips. (**A**,**C**,**E**) show results for gE-2 mutant heterodimers #1–8. (**B**,**D**,**F**) show results for gE-2 mutant heterodimers #9–15. gE-1 monomer included as a positive control. A protein that does not associate with IgG Fc is included as a negative control. Association phase shown.

Our next objective was to leverage the knowledge of the gE-2/gI-2 heterodimer:IgG Fc interaction for rational vaccine design. We hypothesized that if using wildtype gE-2/gI-2 as a vaccine, the antigen complex might be masked by plasma IgG Fc, especially for the critical Fc binding surface of gE-2 where we would like the elicited antibodies to target. The gE-1:IgG Fc binding interface has been identified by the published co-crystal structure of gE-1/gI-1 with IgG Fc [14]. Several likely Fc-binding stretches were defined within gE-1, including amino acid stretches Ser245-Ala250, Arg316-Ser324, and Glu338-Val342. Alignment of the ectodomains of gE-1 and gE-2 reveal a sequence identity of 71.7% within the ecto domains (Figure 4). Based on this analysis, 16 mutations of gE-2 t417 (strain 333) within the hypothesized Fc:gE-2 interface were generated with the goal of eliminating or reducing Fc binding. The mutant protein was co-expressed with wildtype gI-2 t262 (strain 333). We focused the gE-2 mutagenesis efforts on the putative surface-exposed loop of gE-2 at Ala337-Val340 due to the analogous loop in gE-1 having significant binding interactions with Fc (Table 1). A second series of mutations in gE-2 were made at positions His245, Pro317, and Pro319 owing to the gE-1 equivalent amino acids’ proximities, within 5Å, of Fc in the co-crystal structure (Table 1) [14]. We designed the gE-2 mutants to contain a minimal number of mutations per construct in order to preserve the global and local structure of this critical surface for functional antibodies. Of the 16 gE-2 mutants, four failed to express secreted protein (Table 1), and all other gE-2 mutants were able to be expressed as heterodimers with gI-2. Characterization of the twelve remaining gE-2 mutant heterodimers by SEC-MALS showed both molecular weights and hydrodynamic radii comparable to the wildtype gE-2/gI-2 heterodimers, suggesting preservation of the global structure (Table 2, lines 10–21 vs. line 6). Evaluation of the gE-2 mutant heterodimers in the IgG Fc binding SPR assay identified nine gE-2 mutations that abrogated or reduced the ability of the gE-2/gI-2 heterodimer to associate with human and rabbit IgG Fc: a 4 amino acid insertion at position A337 (mutant # 1); T339G (mutant # 4); S338G +T339G (mutant # 6); S338G + T339G + V340G (mutant # 9); H245G (mutant # 10); P317G (mutant # 11); P319G (mutant #12); H245G + P317G (mutant # 13); and P317G + P319G (mutant # 15) (Table 1 and Table 4, Figure 5). The remaining three mutations did not affect heterodimer association with IgG Fc.

### 3.6. Antibodies Elicited by gE-2/gI-2 Heterodimers Deficient in Binding IgG Fc Neutralize HSV-2 Virus Better than Those Elicited by Wildtype gE-2/gI-2 Heterodimers

**Figure 6:**
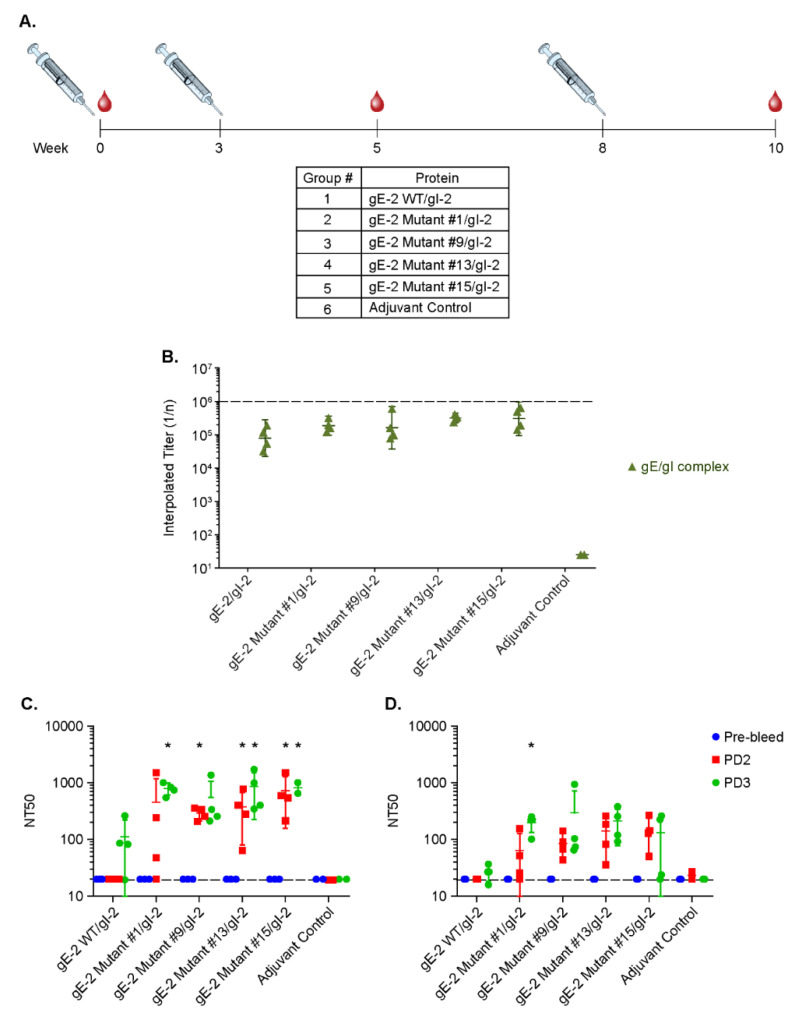
Rabbit immunogenicity study of gE-2/gI-2 mutant heterodimers. (**A**) In vivo immunogenicity study outline. Syringe icon denotes immunization. Blood drop icon indicates serum collection. (**B**) Serum antibody ELISA titers against gE-2/gI-2 wildtype heterodimer. (**C**) Serum neutralization antibody titers in the presence of complement. gE-2/gI-2 mutant heterodimer groups whose response is statistically significant (*p* < 0.05) over wildtype gE-2/gI-2 heterodimer are noted with an asterisk (*). (**D**) Serum neutralization antibody titers in the presence of complement and non-specific IgG. Virus was pre-incubated with 10 mg/mL non-specific rabbit IgG for 1 h prior to addition of serum samples. gE-2/gI-2 mutant heterodimer groups whose response is statistically significant (*p* < 0.05) over wildtype gE-2/gI-2 heterodimer are noted with an asterisk (*).

The ability of gE to bind IgG Fc is a putative mechanism of HSV immune evasion. To evaluate the changes in antibody responses elicited by mutant gE-2/gI-2 heterodimers compared to the wildtype heterodimers, we needed to select an appropriate animal species whose IgG interacts with the wildtype gE-2/gI-2. We chose to use rabbit as the animal model, as our data show that the gE-2/gI-2 complex binds rabbit IgG similarly to human IgG, much better than IgGs from guinea pig and mouse (Figure 3C–E red trace and Table 3 line 7). We selected four gE-2/gI-2 heterodimers that appeared most deficient in associating with IgG Fc (#1, #9, #13, and #15) and evaluated them in a rabbit immunogenicity study. The rabbits received three doses of 20 μg of Adju-Phos adjuvanted protein vaccine at weeks 0, 3, and 8, and immune sera were collected at weeks 0, 5, and 10 (Figure 6A). Serum antibody binding titers against wildtype gE-2/gI-2 heterodimer are similar for all the mutant gE-2/gI-2 heterodimers and are equal to or better than titers elicited by the wildtype gE-2/gI-2 heterodimer (Figure 6B). The serum neutralization antibody titers were higher for the Fc-binding deficient heterodimers compared to wild-type heterodimers. At post dose 2, detectable titers were observed for all mutant groups but not for the wildtype, and statistically significant differences were observed for mutants #9, #13, and #15 compared to wildtype. At post dose 3, titers from the mutant groups are up to 10-fold higher compared with the wildtype group, with statistically significant differences observed for mutants #1, #13, and #15 compared to wildtype (Figure 6C). To determine the neutralization potential generated by the wildtype and mutant gE-2 heterodimers under conditions more analogous to in vivo settings, we pre-incubated virus with a physiological concentration of non-specific IgG (10 mg/mL) prior to serum dilution and repeated the virus neutralization assay (Figure 6D). The serum neutralization antibody levels following virus pre-incubation trended higher for the Fc-binding deficient heterodimers when compared to wildtype heterodimers. At post dose 2, detectable titers were observed for all mutant groups but not for the wildtype. At post dose 3, titers from the mutant groups are at least 2-fold higher compared with the wildtype group, with statistically significant differences observed for mutant #1 compared to wildtype.

## 4. Discussion

We have described expression and purification of three soluble gE-2 truncates as well as the co-expression and purification of twelve soluble gE-2/gI-2 mutants. The gE-2 variants are based on sequences from several HSV-2 strains and have different truncations at the N and C termini. We found that none of the three gE-2 variants expressed in mammalian cells associate with either whole-molecule IgG or IgG Fc as a monomer regardless of ectodomain truncation position, viral strain amino acid sequence, or species of IgG Fc (Figure 3 and Table 3). This contrasts with a previously published report showing a gE-2 protein truncated at amino acid position 405 and purified from baculovirus-infected Sf9 insect cells binds to IgG Fc of several species in the absence of gI-2 [40]. In our study, binding to IgG Fc is observed once gE-2 is heterodimerized with gI-2 (Figure 3, Table 3). While we do not have a clear understanding of the apparent discrepancy, we do note that the insect cell expression system, one-step affinity chromatography purification method, ELISA assay system, and IgG source were all different in the previously published report compared to our study presented here. These factors could all play a part and account for the differences observed. Previous studies have shown that while gE-1 is capable of binding to IgG Fc alone, gE-1/gI-1 heterodimerization increases binding affinity by 50–100 fold [13,14]. The requirement of the formation of gE-2/gI-2 complex for IgG binding observed here, taken together with previous data on gE-1/gI-1 showing increased IgG Fc-binding, suggests that gI plays an important role within the gE/gI heterodimer with respect to IgG Fc binding for both HSV-1 and HSV-2. gI may induce conformational changes within gE upon complex formation and thereby increase its affinity for IgG Fc. Additionally, gI may stabilize the gE structure so that the IgG binding site is better exposed or better positioned for IgG Fc binding. Finally, gI may make important contacts with IgG Fc, which are not sufficient for binding Fc on its own but stabilize the gE/IgG Fc interactions and thereby enable binding to gE directly. Additional experiments are required to elucidate the contributions of each of these potential mechanisms.

**Figure 7:**
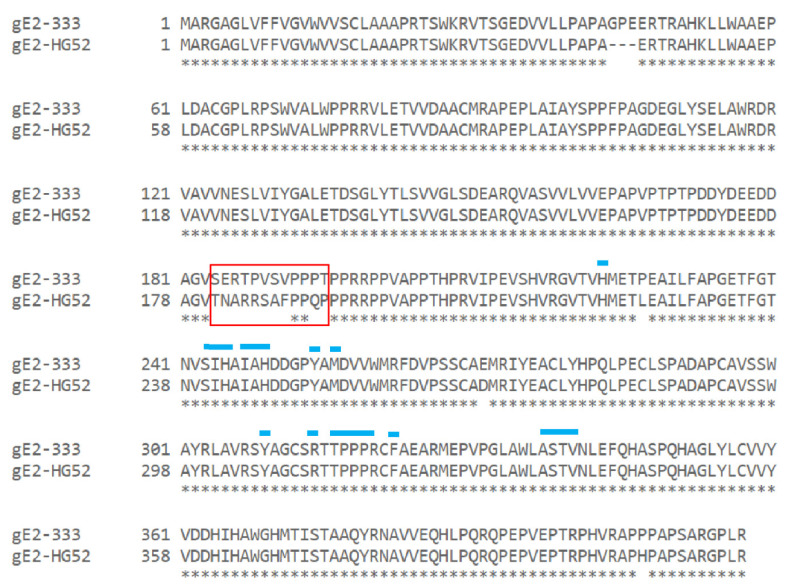
Sequence alignment of HSV-2 gE ecto domains. Alignment of the ecto domains of HSV-2 gE from strains 333 and HG52 shows a 96.2% sequence identity. The red box indicates regions of significant strain variation. Residues within gE-1 predicted to make direct contact with IgG Fc, based on the published co-crystal structure [14], are denoted by blue lines above the sequence.

We showed here that gE-2/gI-2 association with IgG Fc can be affected by ectodomain truncation position within gE-2 and by the viral strain from which the gE-2 sequence is derived. Truncation of the gE-2 ectodomain at position 405 versus immediately prior to the transmembrane regions at position 417 reproducibly improves association with Fc (Figure 3 red vs. light green trace and Table 3 line 7 vs. 8). As the Fc-interacting residues are hypothesized to lie toward the C-terminus of the gE ectodomain, removal of amino acid residues proximal to this site may make the binding surface more accessible to Fc or allow for a tighter binding due to alleviation of steric hinderance by portions of gE-2 itself. The strength of IgG Fc binding to gE-2/gI-2 also depends on the strain from which the gE-2 sequence was derived. Alignment of the gE-2 ectodomain of strain 333 and HG52 reveals a 96.2% sequence identity (Figure 7). The known Fc binding site on gE is highly conserved between the two strains; however, a further investigation into the alignment reveals a stretch of poor homology in gE-2 strains 333 and HG52 between amino acids 184 through 195 of strain 333 (AA181-AA192 for HSV-2 strain HG52). The amino acids in this N-terminal region of gE-1 have been shown to associate with gI and thus play a role in gE/gI heterodimer formation [18]. This stretch of non-homologous amino acids may be more optimal in the HSV-2 HG52 strain for association with gI, leading to a more stable or more functional heterodimer than the heterodimer of HSV-2 strain 333. Swapping this amino acid stretch between the two HSV-2 strains or comparing their thermal melt profiles could provide additional insight into heterodimer formation, stability, and subsequent IgG Fc association.

To evaluate HSV-2 gE/gI as a potential vaccine candidate, we needed to select an animal species whose IgG interacts with the gE/gI complex comparable to the human interaction. The functional distinction of the gE/gI heterodimer for IgG from different species is critical for the selection of an appropriate animal model and the interpretation of data generated from that model. Indeed, we showed here that gE-2/gI-2 association with IgG Fc is affected by the species of IgG Fc. The gE-2/gI-2 heterodimer strongly (highest RU amplitude) associates with human and rabbit IgG. Definitive gE-2/gI-2 heterodimer binding was not observed for guinea pig IgG, though weak binding to guinea pig IgG was observed for gE-1 and the gE-1/gI-1 heterodimer (Table 3). No positive association with mouse IgG is seen for either gE-1 or gE-2 regardless of truncation position, HSV strain, or heterodimerization state (Figure 3D and Table 3). The primary animal challenge models used in the field of HSV-2 virology and vaccinology include mouse and guinea pig. Our data suggest that vaccines containing gE-2, which are evaluated in guinea pig and mouse models, species whose IgG Fc does not associate well with gE-2, might not generate data that can appropriately evaluate the contribution of gE-2 to the vaccine. Further, studies in mouse and guinea pig models may underestimate the impact of gE-2 inclusion in an HSV vaccine due to the inability to effectively evaluate effects upon immune evasion through IgG Fc association. Instead, the contribution of a gE-2 or gE-2/gI-2 heterodimer antigen to protection from infection or prevention of disease would more appropriately be evaluated by developing an HSV-2 challenge model in the rabbit, where IgG Fc association is comparable to human.

**Figure 8:**
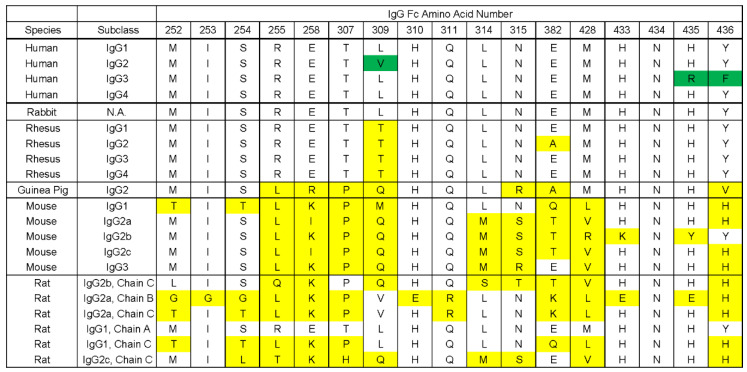
Sequence alignment of IgG Fc hinge region residues from various animal species. Alignment of the residues within the hinge region of IgG from multiple species that have been defined as playing a role or being important for gE-1/gI-1 interaction and/or binding contact. Green highlighting indicates a different amino acid from the most common at that position for subclasses of human or rabbit IgG. Yellow highlighting indicates a discrepancy between that species’ sequence and the analogous human/rabbit amino acid in that position.

The IgG Fc species specificity data parallel the work done for HSV-1, where HSV-1 infected cells were probed for their ability to bind iodinated human and animal immunoglobulins. It was found that the best association was seen with human and rabbit IgG, while little to no association was seen to rodent IgG, including guinea pig and mouse [19]. Several studies elucidated the residues within IgG Fc, which contributed most to the interaction with the HSV gE/gI heterodimer [13,14]. A binding “hot spot” of residues within the hinge region of IgG Fc was described [41,42]. Of particular importance is residue histidine 435 within the Fc hinge region [20,43]. Figure 8 shows an alignment of IgG from various species across the key residues thought to be involved in gE/gI heterodimer binding by the IgG Fc. Of note, all subclasses of human IgG and the rabbit IgG have identical or nearly identical residues across all critical amino acid positions, while many differences were found in other species compared to human. This sequence identity supports the data observed here, that human and rabbit IgGs show association with HSV-1 and HSV-2 gE/gI heterodimers to the highest level, as indicated by the RU amplitude in the SPR assay. Of the species interrogated by this study, guinea pig IgG has the next fewest changes; 7 out of 17 amino acid residues are different from that of human. This could account for the significant decrease in IgG association by HSV of any strain or type of gE/gI. Finally, mouse IgG shows 8–10 amino acid differences as compared to human IgG, depending on subclass, in this critical IgG Fc hinge region, including one subclass having a change at His435 position. This number of changes appears not to be tolerated by either gE-1 monomer or HSV-1/2 HSV-2 gE/gI heterodimer. Based on these data and the IgG sequence alignments, we hypothesize that the gE/gI heterodimer would associate well with rhesus IgG but poorly, if at all, with that of rat.

With these data on HSV-2 gE/gI function, we next sought to exploit the knowledge of the gE-2/gI-2 heterodimer:IgG Fc interaction for rational vaccine design. Anti-gE-specific antibodies that disrupt Fc binding may mitigate immune evasion and thereby elicit an immune response absent in previous HSV-2 vaccine development. We hypothesize that wildtype gE-2/gI-2 antigen might be masked by a non-specific host IgG shield decorating gE via the Fc domain. This “shield” would render wildtype gE epitopes inaccessible to the immune system, decreasing the specific gE antibody response and rendering the gE antigen less immunogenic. Engineering a gE-2 molecule that is unable to associate with IgG Fc would eliminate this IgG shield and expose the critical gE-2:Fc interface as a new antigenic region. These now unshielded mutant antigens might be able to elicit a better neutralizing antibody response that targets the Fc binding surface and disrupts the immune evasion function of HSV gE. Evaluation of twelve gE-2 mutant heterodimers in the IgG Fc binding SPR assay identified nine gE-2 mutations that abrogated or greatly reduced human Fc binding. These data suggest a critical role for IgG Fc interaction within the putative surface-exposed loop ASTV_340_ in gE-2. Based on the current mutations within this loop, V340 appears to play the most critical role in IgG Fc association with gE-2. A more puzzling result is that for heterodimer mutant #8, having a triple glycine substitution at positions A337 + S338 + T339, which shows little to no effect on IgG Fc binding (Table 4, Figure 5A,C,E dark red trace). Single point mutation at S338 also shows no effect of gE-2/gI-2 heterodimer binding IgG Fc. However, single substitution at T339 and double substitution of S338 + T339 results in decreased Fc binding. Addition of the A337G mutation to the S338 + T339 double mutant appears to restore Fc binding. One possibility is that the addition of this mutation might restore a conformation or create a more permissible local environment for binding of IgG Fc. Additional single point mutations of A337G and double point mutations at positions A337 and T339 may shed light on the role of the residues and this surface loop region in Fc binding. For the IgG Fc proximal residues, our data suggest that residues H245, P317, and P319 may serve to function in concert. Single alteration of these residues does not completely abrogate Fc binding to the gE-2/gI-2 heterodimer, but alteration of two or more of these residues results in either lack of heterodimer protein expression or abrogation of Fc binding by the heterodimer (Table 1 and Table 4, Figure 5).To assess the immunogenicity of these four Fc-binding deficient gE-2/gI-2 heterodimers, we evaluated them in an in vivo immunogenicity experiment. Based on our data showing robust binding of gE-2/gI-2 heterodimers to rabbit IgG (Figure 6A), we selected rabbit as our model system. In vivo, the Fc-binding deficient heterodimers elicited comparable binding antibody titers to wildtype gE-2/gI-2 heterodimers, suggesting that there are no apparent quantitative differences in the immune responses (Figure 6B). Differentiation between our Fc-binding deficient heterodimers and wildtype heterodimers was observed in the ability of the immune serum to neutralize HSV-2 virus. Fc-binding deficient heterodimers elicited statistically significant, higher serum neutralization antibody levels than wildtype heterodimers (Figure 6C). We sought to further examine the apparent superior neutralizing antibody response elicited by our Fc-binding deficient heterodimers by mimicking a human in vivo environment, as human serum contains 6–16 mg/mL of IgG [44]. Fc-binding deficient heterodimers elicited neutralizing antibody titers that trended higher than neutralization antibody levels elicited from wildtype heterodimers when virus was preincubated with 10 mg/mL rabbit total IgG (Figure 6D), with the gE-2 mutant #1/gI-2 heterodimer having statistically significant, higher neutralizing antibody titers. SPR data suggest that human and rabbit IgG dissociates rapidly from gE-2/gI-2 heterodimers (Figure 3B–C and Table 3). In vivo, this dissociation profile may allow opportunity for antibodies elicited and directed against the Fc-binding surface of gE-2 to associate with the wildtype viral glycoprotein heterodimer, even in the presence of physiologically relevant serum IgG. In total, these data show that while the quantity of the antibody response is equivalent between the mutant and wildtype heterodimers, the quality of the elicited anti-gE/gI antibodies indeed improved when the gE-2 in the vaccine was unable to bind Fc.

It is important to note -our data showed that gE-2 alone is incapable of binding IgG Fc. It is therefore possible that gE-2 alone may not be masked by serum IgG nor benefit from targeted mutagenesis described here for the heterodimer vaccines. However, the lack of detectable IgG Fc binding by gE-2 could indicate an altered global conformation, unstable structure, or a less exposed IgG Fc binding surface of the gE-2 monomer protein. While these hypotheses could contribute potentially to an overall diminished contribution of neutralizing antibody titers by gE-2 alone, experiments comparing quantity and quality of immune response generated by gE-2 alone and the mutant gE-2/gI-2 heterodimer could shed additional light on the nuances of gE-2/gI-2 heterodimer function and the comparative advantage of gE-2 and gE-2/gI-2 to function as vaccine antigen candidates.

We have shown that gE-2 requires heterodimerization with gI-2 to bind to IgG Fc. HSV gE-2 association with IgG Fc can be modulated by the length of truncated gE, HSV strain, and species of IgG. Our data support the hypothesis that IgG Fc binding of gE plays an important role in immune evasion, and the abrogation of this interaction with functional antibodies enables increased protection from viral infection. We established a proof of concept showing that rationally designed HSV antigens with reduced host protein binding elicit improved functional antibody responses compared to wildtype antigens, including in IgG serum environments that mimic in vivo conditions. Exploration of this strategy and the presented constructs in further preclinical studies will help to fully understand the potential of these newly designed gE-2 vaccine candidates.

## 5. Conclusions

Herpes simplex virus 2 is the primary cause of genital ulcers in both the United States and worldwide. Currently, no vaccine exists for HSV. Most vaccine approaches against HSV-2 have focused on viral surface glycoproteins. A better understanding of the functions and the associated functional domains of these viral glycoproteins will enable engineering of improved antigens and thereby lead to superior vaccines. Here we show that IgG Fc binding of HSV-2 gE requires the formation of the gE/gI heterodimer to bind IgG-Fc, and that binding varies by species of IgG-Fc. We designed functionally deficient gE-2/gI-2 heterodimers and showed that vaccination with these mutant heterodimers elicits higher neutralizing antibody titers against HSV-2 than wildtype heterodimers both without and in the presence of physiological serum IgG levels, thus highlighting the potential of rational vaccine design for HSV.

## Figures and Tables

**Table 1:** Mutations within HSV-2 gE designed to disrupt gE2/IgG Fc binding. Mutations of gE-2 strain 333 were localized to two regions. The first set of mutations was made based on the putative surface-exposed loop at A337-V340 (top section of table). Mutations of gE-2 strain 333 were also made based on a series of positions hypothesized to be proximal to the IgG Fc binding interface: positions H245, P317, and P319 (bottom section of table). Protein expression data from transient transfection of mammalian cells are summarized.

	Construct	Amino Acid Number (Based on gE-2 Sequence)				Mutation Type	Obtained Protein Expression (Y/N)
		337	338	339	340		
Putative Surface-exposed Loop Mutations	**gE-2t 417 WT**	**A**	**S**	**T**	**V**	**N.A.**	**Y**
	gE-2t 417 Mutant # 1	AARAA	S	T	V	Insertion	Y
	gE-2t 417 Mutant # 2	A	G	T	V	Substitution	Y
	gE-2t 417 Mutant # 3	A	-	T	V	Deletion	N
	gE-2t 417 Mutant # 4	A	S	G	V	Substitution	Y
	gE-2t 417 Mutant # 5	A	S	-	V	Deletion	Y
	gE-2t 417 Mutant # 6	A	G	G	V	Substitution	Y
	gE-2t 417 Mutant # 7	A	-	-	V	Deletion	N
	gE-2t 417 Mutant # 8	G	G	G	V	Substitution	Y
	gE-2t 417 Mutant # 9	A	G	G	G	Substitution	Y
		245	317	319			
Putative Proximal IgG Fc Binding Interface Mutations	**gE-2t 417 WT**	**H**	**P**	**P**		**N.A.**	**Y**
	gE-2t 417 Mutant # 10	G	P	P		Substitution	Y
	gE-2t 417 Mutant # 11	H	G	P		Substitution	Y
	gE-2t 417 Mutant # 12	H	P	G		Substitution	Y
	gE-2t 417 Mutant # 13	G	G	P		Substitution	Y
	gE-2t 417 Mutant # 14	G	P	G		Substitution	N
	gE-2t 417 Mutant # 15	H	G	G		Substitution	Y

**Table 2:** SEC MALS analysis of HSV gE monomers and gE/gI heterodimers. The monomer and heterodimers in the upper part of the table contain all wildtype sequences (lines 1–9). The heterodimers in the bottom part of the table contain mutations within the gE-2 monomer (lines 10–21).

Line #	Protein	Strain	Theoretical Molecular Weight (Da)	Apparent Molecular Weight (Da)	Apparent Molecular Weight Carbohydrate (Da)	Hydrodynamic Radius (nm)
1	Bac gE-1 t411	17	42,817	53,390	N.A.	5.556
2	gE-1 t419	KOS	44,273	44,462	6847	4.512
3	gE-2 t417	333	44,795	49,907	17,717	5.622
4	gE-2 t-405	333	43,597	42,428	15,667	5.011
5	gE-1 t419/gI-1 t269	KOS	72,408	70,073	28,146	5.957
6	gE-2 t417/gI-2 t262	333	72,169	69,905	26,006	5.446
7	gE-2 t414/gI-2 t262	HG52	72,012	69,167	28,374	5.526
8	gE-2 t405/gI-2 t262	333	70,971	67,927	21,985	5.087
9	gE-2 t24-405/gI-2 t262	333	70,008	66,664	25,057	5.149
10	gE-2 t417 Mutant #1/gI-2 t262	333	72,538	68,290	28,414	5.427
11	gE-2 t417 Mutant #2/gI-2 t262	333	72,139	68,020	27,977	5.396
12	gE-2 t417 Mutant #4/gI-2 t262	333	72,124	69,350	26,852	5.474
13	gE-2 t417 Mutant #5/gI-2 t262	333	72,067	69,582	28,452	5.398
14	gE-2 t417 Mutant #6/gI-2 t262	333	72,094	68,749	26,822	5.33
15	gE-2 t417 Mutant #8/gI-2 t262	333	72,080	69,444	25,675	5.222
16	gE-2 t417 Mutant #9/gI-2 t262	333	72,052	69,105	26,540	5.236
17	gE-2 t417 Mutant #10/gI-2 t262	333	72,088	69,563	26,205	5.312
18	gE-2 t417 Mutant #11/gI-2 t262	333	72,128	70,121	26,437	5.318
19	gE-2 t417 Mutant #12/gI-2 t262	333	72,128	69,323	27,000	5.309
20	gE-2 t417 Mutant #13/gI-2 t262	333	72,048	70,327	28,454	5.395
21	gE-2 t417 Mutant #15/gI-2 t262	333	72,088	69,574	26,905	5.336

**Table 3:** Binding of gE-2 monomer and gE-2/gI-2 heterodimer to IgG Fc: SPR analysis. Association of gE monomers and gE/gI heterodimers to multiple species of IgG was determined by SPR. gE-1 monomers and gE-1/gI-1 heterodimer are included as positive controls. A protein that does not associate with IgG Fc is included as a negative control. Summary of response units (RU) at the end point of the association phase and dissociation phase (shown in parentheses). IgG is whole molecule IgG except where noted as the Fc domain only (IgG Fc).

Line #	Protein	Virus Strain	Expression System	RU Post Association (RU Post Dissociation)				
				Human IgG Fc	Human IgG	Rabbit IgG	Mouse IgG	Guinea Pig IgG
1	Bac gE-1 t411	17	Insect	77 (12)	147 (32)	231 (75)	4 (−3.1)	32 (3.3)
2	gE-1 t419	KOS	Mam.	69 (1.2)	127 (−1.9)	188 (0.6)	4 (−4.0)	27 (−2.4)
3	gE-1 t419/gI-1 t269	KOS	Mam.	216 (137)	232 (189)	195 (170)	11 (−2.3)	19 (0.8)
4	gE-2 t417	333	Mam.	5 (0.2)	4 (−2.7)	11 (−2.7)	10 (−2.7)	5 (−0.7)
5	gE-2 t405	2.12	Mam.	5 (0.2)	8 (−1.7)	6 (−1.8)	4 (−4.0)	5 (−1.4)
6	gE-2 t24-405	333	Mam.	8 (1.2)	7 (−2.2)	6 (−1.2)	4 (−3.8)	5 (−0.03)
7	gE-2 t417/gI-2 t262	333	Mam.	41 (0.8)	52 (−2.1)	46 (−1.5)	10 (−2.0)	9 (−0.07)
8	gE-2 t405/gI-2 t262	333	Mam.	100 (7.7)	142 (14)	165 (34)	8 (−3.5)	9 (−1.5)
9	gE-2 t24-405/gI-2 t262	333	Mam.	95 (6.6)	136 (12)	142 (25)	6 (−4.2)	8 (−1.9)
10	gE-2 t414/gI-2 t262	HG52	Mam.	53 (0.6)	79 (−0.4)	75 (−0.6)	5 (−3.8)	4 (−2.1)
11	Negative Control Protein	N.A.	Mam.	4 (−1.3)	2 (−2.2)	11 (−2.8)	4 (−3.5)	2 (−1.8)

**Table 4:** Fc binding of gE-2 mutants: SPR analysis. Maximal association response at the end of the association phase is shown. gE-1 monomer is included as a positive control. A protein that does not associate with IgG Fc is included as a negative control. Mutations that abrogated or decreased IgG Fc binding are in red (Mam: mammalian). IgG is whole molecule IgG except where noted as the Fc domain only (IgG Fc).

	Protein	Virus Strain	Expression System	RU Post Association		
				Human IgG Fc	Human IgG	Rabbit IgG
Run 1	gE-1 t411	17	Insect	80	145	221
	gE-2t 417/gI-2 t262	333	Mam.	98	133	138
	gE-2 t417 Mutant # 1/ gI-2 t262	333	Mam.	5	1	3
	gE-2 t417 Mutant # 2/ gI-2 t262	333	Mam.	89	121	125
	gE-2 t417 Mutant # 4/gI-2 t262	333	Mam.	18	23	37
	gE-2 t417 Mutant # 5/gI-2 t262	333	Mam.	60	82	72
	gE-2 t417 Mutant # 6/gI-2 t262	333	Mam.	9	8	11
	gE-2 t417 Mutant # 8/gI-2 t262	333	Mam.	102	134	87
	Negative Control Protein	N.A.	Mam.	2	4	1
Run 2	gE-1 t411	17	Insect	80	157	228
	gE-2t 417/gI-2t 262	333	Mam.	43	53	45
	gE-2 t417 Mutant # 9/gI-2 t262	333	Mam.	4	2	4
	gE-2 t417 Mutant # 10/gI-2 t262	333	Mam.	18	19	23
	gE-2 t417 Mutant # 11/gI-2 t262	333	Mam.	19	18	21
	gE-2 t417 Mutant # 12/gI-2 t262	333	Mam.	27	32	62
	gE-2 t417 Mutant # 13/gI-2 t262	333	Mam.	5	3	5
	gE-2 t417 Mutant # 15/gI-2 t262	333	Mam.	8	5	4
	Negative Control Protein	N.A.	Mam.	1	3	2

## Data Availability

The data presented within this study are available within the manuscript.
